# Characterization of a copper transporter 1 from *Dermanyssus gallinae* as a vaccine antigen

**DOI:** 10.1017/S0031182021001608

**Published:** 2022-01

**Authors:** Sotaro Fujisawa, Shiro Murata, Masayoshi Isezaki, Takuma Ariizumi, Takumi Sato, Eiji Oishi, Akira Taneno, Naoya Maekawa, Tomohiro Okagawa, Osamu Ichii, Satoru Konnai, Kazuhiko Ohashi

**Affiliations:** 1 Department of Disease Control, Faculty of Veterinary Medicine, Hokkaido University, Sapporo, Japan; 2 Department of Advanced Pharmaceutics, Faculty of Veterinary Medicine, Hokkaido University, Sapporo, Japan; 3 Vaxxinova Japan K.K., Tokyo, Japan; 4 Department of Basic Veterinary Science, Faculty of Veterinary Medicine, Hokkaido University, Sapporo, Japan; 5 Laboratory of Agrobiomedical Science, Faculty of Agriculture, Hokkaido University, Sapporo, Japan

**Keywords:** Copper transporter 1, *Dermanyssus gallinae*, Dg-Ctr1, poultry red mite, vaccine

## Abstract

Poultry red mites (*Dermanyssus gallinae*, PRM) are dangerous ectoparasites that infest chickens and threaten the poultry industry worldwide. PRMs usually develop resistance to chemical acaricides, necessitating the development of more effective preventive agents, and vaccination could be an alternative strategy for controlling PRMs. The suitability of plasma membrane proteins expressed in the midguts as vaccine antigens was evaluated because these molecules are exposed to antibodies in the ingested blood and the binding of antibodies could potentially induce direct damage to midgut tissue and indirect damage *via* inhibition of the functions of target molecules. Therefore, in the present study, a copper transporter 1-like molecule (Dg-Ctr1) was identified and its efficacy as a vaccine antigen was assessed *in vitro*. *Dg-Ctr1* mRNA was expressed in the midguts and ovaries and in all the life stages, and flow cytometric analysis indicated that Dg-Ctr1 was expressed on the plasma membrane. Importantly, nymphs fed on plasma derived from chickens immunized with the recombinant protein of the extracellular region of Dg-Ctr1 showed a significant reduction in the survival rate. These data indicate that the application of Dg-Ctr1 as a vaccine antigen could reduce the number of nymphs in the farms, contributing to reduction in the economic losses caused by PRMs in the poultry industry. To establish an effective vaccination strategy, the acaricidal effects of the combined use of Dg-Ctr1 with chemical acaricides or other vaccine antigens must be examined.

## Graphical abstract

**Figure:**
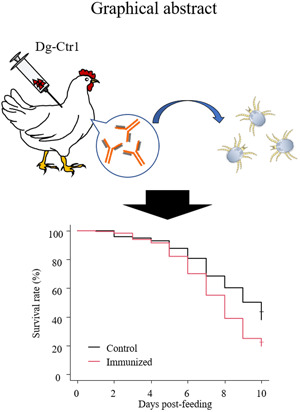

## Introduction

The poultry red mite (*Dermanyssus gallinae*, PRM) is one of the most harmful haematophagous ectoparasites of chickens. The lifecycle of PRMs consists of eggs, larvae, protonymphs, deutonymphs and adults; PRMs typically feed on blood every 2–4 days (Sparagano *et al*., 2014). Mass infestation by PRMs causes various harmful effects, such as anaemia, resulting in significant loss of productivity in poultry farming. The current acaricide-based methods used for PRM prevention are usually insufficient since PRMs hide in cracks and crevices after sucking blood and also develop resistance to the acaricides. Thus, there is an urgent need for alternative PRM control methods.

Vaccination has been recently highlighted as a promising strategy for the control of ectoparasites, including PRMs, and several studies have reported antigen candidates and their efficacies (Bartley *et al*., 2012; Wright *et al*., 2016; Lima-Barbero *et al*., 2019). However, vaccine efficacies have yet to be sufficiently observed in practical application in the field (Bartley *et al*., 2017). Thus, vaccination strategies have to be improved, and evaluation of more effective vaccine antigens is essential to increase the efficacies of these vaccines.

As for the targets of vaccine antigens to prevent haematophagous ectoparasites, two types of antigens could be taken into consideration: the first group includes secreted proteins from salivary glands that facilitate attachment to the host skin and blood sucking by suppressing host immune responses (Willadsen, 1980; Wikel, 1996). The other groups includes antigens expressed in the midguts, because the sucked blood accumulates in the midguts, and the midgut antigens could be efficiently exposed to antibodies present in the blood sucked from immunized animals (Willadsen *et al*., 1989; Pritchard *et al*., 2015). While tick species infest hosts and continue blood feeding for longer periods (3–10 days or more; Ribeiro and Francischetti, 2003), PRMs infest chickens within a few minutes to an hour and do not stay on chicken bodies after sucking blood. In addition, PRMs are intermittent feeders and repetitively suck blood in the life cycle (Pritchard *et al*., 2015). Therefore, midgut proteins might be suitable for development as vaccine antigens against PRMs. In addition, several studies have demonstrated that the immunization of cattle with transmembrane protein BM86 and its orthologs showed remarkable acaricidal effects on the ticks *Boophilus microplus* and *Hyalomma a. anatolicum*, respectively, even though the physiological function of BM86 remains unknown (Rand *et al*., 1989; Azhahianambi *et al*., 2009). Collectively, plasma membrane proteins expressed in the midguts would be desirable as targets of vaccines.

For the development of anti-PRM vaccines, the ideal vaccine antigens should show high expression levels and be constitutively expressed in all life-stages or at least in the blood-fed stages of PRMs, in addition to the above-mentioned characteristics. A comparative analysis of the transcriptome between blood-fed and starved PRMs has been performed previously (Fujisawa *et al*., 2020), and it identified a novel copper ion transporter 1-like transcript (*Dg-Ctr1*) that showed high expression intensities in both blood-fed and starved states. Copper ion transporter 1 plays a role in copper uptake, and the copper ion is an essential nutrient for arthropods, playing critical roles in respiration (Muttkowski, 1921), immunity (Christensen *et al*., 2005; Lu *et al*., 2014), pigmentation (Sugumaran and Barek, 2016), and metal homeostasis and detoxification (Amiard *et al*., 2006; Perez and Noriega, 2014; Rivera-Perez *et al*., 2017). Thus, interruption of copper uptake could disrupt PRM homeostasis, making copper ion transporter 1 a candidate antigen for the development of anti-PRM vaccines.

In the present study, the suitability of Dg-Ctr1 as a vaccine antigen was evaluated on the basis of the following criteria: (1) highly expressed molecule in all life stages or at least in the blood-fed stages, (2) molecule expressed in midguts, (3) molecule expressed on the plasma membrane. Gene-expression analyses revealed that *Dg-Ctr1* was detected in the midgut and was expressed in all life stages, regardless of the feeding state. Flow cytometric analysis confirmed that Dg-Ctr1 was expressed on the plasma membrane of the transfected cells, as predicted by its domain and motif. Finally, the survival rates of PRMs fed on plasma from chickens immunized with recombinant Dg-Ctr1 were assessed to evaluate the efficacy of Dg-Ctr1 as a vaccine antigen. Remarkably, nymphs fed immunized plasma showed a significant reduction in the survival rate. Collectively, anti-PRM vaccines targeting Dg-Ctr1 could potentially be a novel method to control PRMs.

## Materials and methods

### Ethics statement

All animal experiments were approved by the Institutional Animal Care and Use Committee, Hokkaido University (Approval number: 20-0051), and all experiments were performed in accordance with the relevant guidelines and regulations of the Faculty of Veterinary Medicine, Hokkaido University, which has been fully accredited by the Association for Assessment and Accreditation of Laboratory Animal Care International (AAALAC).

### Preparation of PRM samples

#### RNA samples of PRMs

PRMs of various developmental stages and both sexes were obtained from an egg-laying farm in Japan. The dark red, round PRMs were designated ‘blood-fed PRMs’ and collected in 1200-*μ*L extra-long filter tips (WATSON Bio Lab, Tokyo, Japan) within 2 days of PRM collection on the farm. The remaining PRMs were stored in a TubeSpin Bioreactor 600 (TPP Techno Plastic Products AG, Trasadingen, Switzerland); maintained at 25°C in 70% humidity for a 1-week period; and designated ‘starved PRMs.’ The blood-fed and some of the starved PRMs were fixed with 70% ethanol, after which the eggs, larvae, protonymphs, deutonymphs, and adults were segregated under microscopic observation. The residual PRMs were stored at 5°C until use for *in vitro* feeding assays.

#### RNA isolation and cDNA synthesis

PRM samples were suspended with 600 *μ*L of Buffer RLT Plus (RNeasy Plus Mini Kit, Qiagen, Hilden, Germany) and thoroughly homogenized using a 1.5-mL homogenization pestle for a 1.5-mL microcentrifuge tube (Scientific Specialties, Inc., Lodi, CA, USA). Total RNA was isolated using a RNeasy Plus Mini Kit according to the manufacturer's protocols. cDNA was synthesized from the isolated RNA by using PrimeScript Reverse Transcriptase (Takara Bio Inc., Shiga, Japan) with 200 pmol of oligo (dT) 18 primer (Hokkaido System Science, Hokkaido, Japan).

### Characterization of *Dg-Ctr1* in PRMs

#### Identification of the cDNA sequence of the copper transporter 1-like molecule in PRMs

A novel copper transporter 1-like transcript (*Dg-Ctr1*) was identified from the RNA-Seq data (Fujisawa *et al*., 2020, BioSample accession numbers: SAMD00228960, SAMD00229086). To determine the sequence of the open reading frame of *Dg-Ctr1*, the *Dg-Ctr1* gene was amplified with Ex-Taq polymerase (TaKaRa Bio Inc.) using the specific primer set DgCtr1-F and DgCtr1-R (Table 1), and performed sequencing analysis using GenomeLab™ GeXP Genetic Analysis System (Beckman Coulter Inc., Brea, CA, USA). The transmembrane domains of Dg-Ctr1 were predicted by InterProScan v5.32-71.0 (https://www.ebi.ac.uk/interpro/). The phylogenetic tree was constructed with MEGA version X (Kumar *et al*., 2018), using the maximum likelihood method with 1000 bootstrap replicates, and with a JTT matrix-based model (Jones *et al*., 1992) by using a discrete Gamma distribution (+G) and by assuming that a certain fraction of the sites was evolutionarily invariable (+I), to improve the tree topology.

**Table 1.** Primers used for amplification of each gene

Primer		Primer sequences (5′–3′)
For gene cloning and sequencing		
DgCtr1-F	AGGACGGTGCAATTCTCAAC	
DgCtr1-R	GTGTTGGCTGGTTGGTGTTT	
For gene-expression analysis		
DgCtr1-RT-F	TGGACCACAGTAACCACGAA	
DgCtr1-RT-R	GCTAGGCACGAACCAAACAT	
Elf1a1-F	GTCGGTGTCATCAAGTCCGT	
Elf1a1-R	AGGGTCGAGAGTGTAGGGTC	
For characterization of expression on the plasma membrane		
FLAG-DgCtr1-F	CCGGATATCGACTATGTGCCAGACTCGCA	
FLAG-DgCtr1-R	CGGGGTACCTCATCCGCAGTGATCCGAGG	
For preparation of the recombinant protein		
pBIC-DgCtr1-F	**GATGACGATGACAAA**GACTATGTGCCAGACTCGCA	
pBIC-DgCtr1-R	**CATCCTGTTAAGCTT**TTAATTTTGTGTCTTCC ACCCG	
		

Restriction enzyme recognition sites are underlined.

Recombination sites are marked with bold font.

#### Laser-capture microdissection

cDNA synthesis from midguts, ovaries and salivary glands was conducted by Laser-capture microdissection (LCM) as previously described (Ichii *et al*., 2017). Starved PRMs belonging to a mix of developmental stages were fixed in Carnoy solution, embedded in paraffin and cut into 5-*μ*m sections. Sections were mounted on glass slides coated with LCM films (Meiwafosis, Tokyo, Japan) and stained with 1% toluidine blue, and tissues were extracted using MicroBeam Rel.4.2 (Carl Zeiss, Oberkochen, Germany). Total RNA from the tissues was isolated using RNAqueous^®^-Micro Kit (Thermo Fisher Scientific, Waltham, MA, USA) according to the manufacturer's instructions, and cDNA was synthesized with the SuperScript™ First-Strand Synthesis System for RT-PCR (Invitrogen), by using 300 pmol of random hexamer primer (Hokkaido System Science).

#### Gene-expression analysis

The expression of *Dg-Ctr1* in each feeding state, developmental stage, and tissue was examined by RT-PCR with Ex-Taq polymerase (Takara Bio Inc.) using the cDNA samples synthesized from blood-feed PRMs, starved PRMs and tissues collected by LCM. The specific primer set consisting of DgCtr1-RT-F and DgCtr1-RT-R was designed and used (Table 1). As an internal control, *elongation factor 1-α 1*-like gene (*Elf1a1*) was amplified, and the primer set Elf1a1-F and Elf1a1-R was used (Table 1) (Ariizumi *et al*., 2021). The detection of the target genes in cDNA synthesized from salivary glands was performed by nested PCR using the same primer set, because the RNA samples extracted from salivary glands were of low quality.

#### Real-time quantitative RT-PCR

To quantify the expression of *Dg-Ctr1* mRNA at each developmental stage and feeding state of PRMs, quantitative PCR was performed using the cDNA samples from different life stages, except for eggs, and each feeding state with the LightCycler480^®^ System II (Roche Diagnostics, Mannheim, Germany) using TB Green Premix DimerEraser (TaKaRa Bio Inc.) according to the manufacturer's instructions. The *Elf1a1* gene was amplified as the internal control. The primers used for quantitative PCR are shown in Table 1. The cycling conditions consisted of initial denaturation at 95°C for 30 s, followed by 45 cycles of 95°C for 5 s, 60°C for 30 s and 72°C for 30 s. To evaluate the specificities of primer pairs, a final melting curve analysis was performed from 65°C to 95°C at a rate of 0.1°C/s. To generate standard curves for quantification, serial dilutions of T-vector pMD20 (TaKaRa Bio Inc.) containing *Dg-Ctr1* or *Elf1a1* were used. Each sample was tested four times, and the expression of *Dg-Ctr1* mRNA was presented as the ratio obtained by dividing the concentration of the *Dg-Ctr1* mRNA by that of *Elf1a1* mRNA.

#### Flow cytometric analysis

To confirm whether Dg-Ctr1 was a plasma membrane-associated protein, the FLAG epitope-tagged recombinant Dg-Ctr1 protein was generated. The ORF of the *Dg-Ctr1* gene was amplified using the specific primer set FLAG-DgCtr1-F and FLAG-DgCtr1-R (Table 1). The PCR amplicon was digested with *EcoR*V-HF (New England Biolabs) and *Kpn*I-HF (New England Biolabs), and the digested fragment was inserted into the vector pCXN2.1-FLAG, which was kindly donated by Dr Takehiko Yokomizo, Juntendo University, Tokyo, Japan. Then, the plasmid was digested with *Nco*I-HF (New England Biolabs) and *Kpn*I-HF (New England Biolabs), and the digested fragment was sub-cloned into the pIEx™-4 vector (Novagen) (pIEx4-FLAG-Dg-Ctr1). pIEx4-FLAG-Dg-Ctr1 and pIEx4-FLAG (mock) were then transfected into Sf9 insect cells using *Trans*IT^®^-Insect Transfection Reagent (Mirus Bio LLC, Madison, WI, USA), according to the manufacturers’ instructions. Cells were harvested at 72 h after the transfection, and stained with Anti DYKDDDDK Monoclonal Antibody (2H8; TransGenic Inc., Fukuoka, Japan) or a mouse IgG2a isotype control (HOPC-1; SouthernBiotech, Birmingham, AL, USA) for 30 min at 4°C. Cells were then washed three times with phosphate-buffered saline (PBS) containing 1% bovine serum albumin (Sigma-Aldrich) (1% BSA-PBS) and stained with PE-conjugated anti-mouse IgG (H + L) polyclonal antibody for 30 min at 4°C. The stained cells were washed twice with 1% BSA-PBS and analysed immediately by FACS Verse (BD Biosciences San Jose, CA, USA) and FCS Express 4 (*De Novo* Software, Glendale, CA, USA).

### Preparation of chicken-derived immune plasma of Dg-Ctr1

#### Preparation of recombinant Dg-Ctr1-N

The recombinant protein of the N-terminal extracellular domain of Dg-Ctr1 was expressed as a fusion protein with the His-tag (Dg-Ctr1-N-his) by using the BIC system (Takara Bio Inc.). Specific primers containing the homologous recombination sites, pBIC-DgCtr1-F and pBIC-DgCtr1-R, were designed for the expression of Dg-Ctr1-N-his according to the manufacturer's instructions (Table 1). PCR was performed with KOD-Plus-Neo (TOYOBO Co., Ltd., Osaka, Japan) to amplify the N-terminal extracellular domain of Dg-Ctr1 (a position of 1–53), and the fragments were introduced into *Brevibacillus* competent cells (*Brevibacillus* Expression System, Takara Bio Inc.) to integrate into the cloning site of the pBIC4 vector (Takara Bio Inc.) by homologous recombination. The transformed bacteria were cultured in TM medium for 48 h at 32°C. The supernatants were then collected, and the proteins were purified using TALON® Metal Affinity Resins (Clontech Laboratories, Inc., Mountain View, CA, USA). The buffer was replaced with PBS using SnakeSkin™ Dialysis Tubing, 3.5 K MWCO (Thermo Fisher Scientific) overnight at 4°C. To confirm the purity of Dg-Ctr1-N-his, the obtained proteins were lysed in 2 × SDS buffer (125 mm Tris–HCl, pH 6.8, 4% SDS, 10% 2-mercaptoethanol, and 20% glycerol), boiled for 5 min, separated using 15% SDS-polyacrylamide gel, and stained with Coomassie brilliant blue (FUJIFILM Wako Pure Chemical Corporation, Osaka, Japan). The concentration of proteins was determined using Pierce™ BCA Protein Assay Kit (Thermo Fisher Scientific) according to the manufacturer's protocols.

#### Immunization of chickens with Dg-Ctr1-N-his

To prepare the immune plasmas, chickens were immunized with Dg-Ctr1-N-his (Supplemental Fig. 1). The purified Dg-Ctr1-N-his was mixed with light liquid paraffin as the adjuvant (20 *μ*g mL^−1^). An emulsion of PBS and light liquid paraffin was prepared and used as the control. Chickens (Hy-Line Brown) were subcutaneously immunized with 10 or 20 *μ*g of Dg-Ctr1-N-his (*n* = 3, each, IM1-3 and IM4-6, respectively) at 3 weeks of age. Four weeks later, the chickens in the immunized group were immunized with the same doses of Dg-Ctr1-N-his with light liquid paraffin subcutaneously. The heparinized blood was collected 3 weeks after the second immunization, and the immune plasma was isolated from the heparinized blood by centrifugation at 2000 ***g*** for 10 min. As the control, three chickens were subcutaneously immunized with PBS, and plasma was isolated 3 weeks after the second immunization (C1–C3).

#### Enzyme-linked immunosorbent assay (ELISA)

Antibody titres in the immune plasmas were determined by ELISA. The purified Dg-Ctr1-N-his was coated on the wells of 96-well plates (Sumitomo Bakelite Co., Ltd, Tokyo, Japan) (100 ng well^−1^) for 16 h with carbon-bicarbonate buffer. After washing each well three times with PBS, PBS containing 0.05% Tween-20 (PBS-T) with 1% BSA was added and incubated at 37°C for 2 h. Wells were then washed five times with PBS-T, and immune plasmas diluted 2000×, 4000×,8000×, 16 000×, 32 000× and 64 000× with PBS were added. After 1 h of incubation at room temperature, the wells were washed five times with PBS-T and incubated with anti-chicken IgY peroxidase rabbit antibody (Sigma-Aldrich) for 1 h at room temperature. Finally, the wells were washed five times with PBS-T and reacted with the TMB one-component substrate (Bethyl Laboratories, Montgomery, TX, USA) for 20 min at room temperature in the dark. The reaction was quenched with 0.18 m H_2_SO_4_ and the absorbance was measured at 450 nm. The assay was performed in duplicate.

#### Western blotting

The production of specific antibodies against Dg-Ctr1-N-his was examined by western blotting. Purified Dg-Ctr1-N-his was separated by using 15% SDS-polyacrylamide gel and then transferred to the polyvinylidene difluoride membranes (Merck Millipore, Burlington, MA, USA). The membrane was blocked overnight at 4°C with PBS-T containing 1% skim milk. The membranes were incubated at room temperature with the isolated immune plasmas, washed three times with PBS-T and incubated at room temperature with anti-chicken IgY peroxidase rabbit antibody (Sigma-Aldrich). Finally, the membranes were incubated with Immobilon Western Chemiluminescent HRP Substrate (Merck Millipore) to visualize the peroxidase signal.

### *In vitro* feeding assay

Fresh chicken blood was collected from healthy chickens maintained at the Field Science Center for Northern Biosphere, Hokkaido University and incubated at 40°C before use. After centrifugation at 2000 ***g*** for 10 min, the plasmas were replaced with an equal volume of the immune plasmas described above. The artificial feeding systems were adopted from Ariizumi *et al*. (2021). Approximately 100 starved PRMs of mixed developmental stages were collected in each feeding device, and the devices were capped with type 2 rubber caps (GE healthcare). For ventilation, the rubber cap was penetrated with a 27 G needle (TERUMO CORPORATION, Tokyo, Japan). Blood feeding was performed for 4 h at 40°C in a dark, humid condition with moderate shaking. Then, only blood-fed PRMs were collected in Pasteur pipettes (day 0) and kept at 25°C in 70% humidity during the observation period. The numbers of dead PRMs were monitored for 10 days, and the anti-PRM property of Dg-Ctr1-N-his immunization was evaluated based on the survival rate as follows:
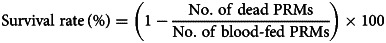


The feeding assay was performed two times: In the first experiment, only nymphs were obtained. In the second experiment, adults and nymphs were separately collected and their survival rates were analysed independently and pooled for analysis.

### Statistics

Differences in qRT-PCR findings were analysed using Mann–Whitney *U* test. To compare PRM mortality between the immunized and control groups after *in vitro* feeding, Kaplan–Meier curves were generated and a log-rank test was performed. Additionally, between-group comparisons of the mortality of PRMs were performed on each day by using Fisher's exact test. The odds ratio and 95% confidence interval (CI) were estimated. *P* values of <0.05 and <0.01 were considered statistically significant.

## Results

### Cloning and sequence analysis of a copper transporter 1-like molecule in PRMs

**Fig. 1.**
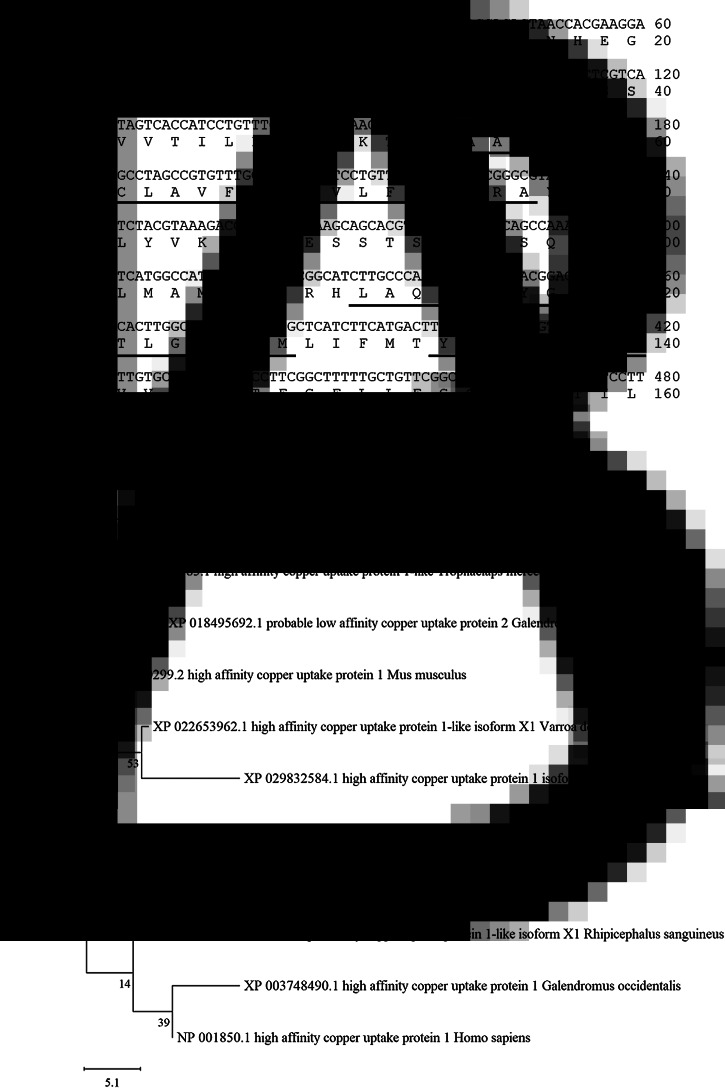
Determination and phylogenetic tree analysis of a *Dermanyssus gallinae* copper transporter 1-like molecule (Dg-Ctr1). (A) Nucleotide sequences and deduced amino acid sequences of Dg-Ctr1. Putative transmembrane sites were underlined. (B) A phylogenic tree based on the deduced amino acid sequence of *Dg-Ctr1*. The tree was built with the maximum likelihood method using the MEGA X software (Kumar *et al*., 2018). Numbers indicate bootstrap percentage (1000 replicates). The scale indicates the divergence time.

The transcript of a copper transporter 1-like molecule with high expression intensities in both blood-fed and starved PRMs was obtained from RNA-Seq analysis data (Fujisawa *et al*., 2020) and designated as Dg-Ctr1. The expression intensity of *Dg-Ctr1* was significantly higher in starved PRMs, but the expression in blood-fed PRMs was also high (Supplemental Table 1). The deduced amino acid sequence of *Dg-Ctr1* contained three putative transmembrane domains at positions 54–76, 110–127 and 133–152 and a large extracellular domain at the N-terminal position 1–53 (Fig. 1A). Phylogenetic analysis using copper transporter 1 of chicken, mammals, ticks and mites revealed that Dg-Ctr1 was closer to copper transporters of mites and ticks rather than those of chickens and mammals (Fig. 1B).

### Dg-Ctr1 expression profile

**Fig. 2.**
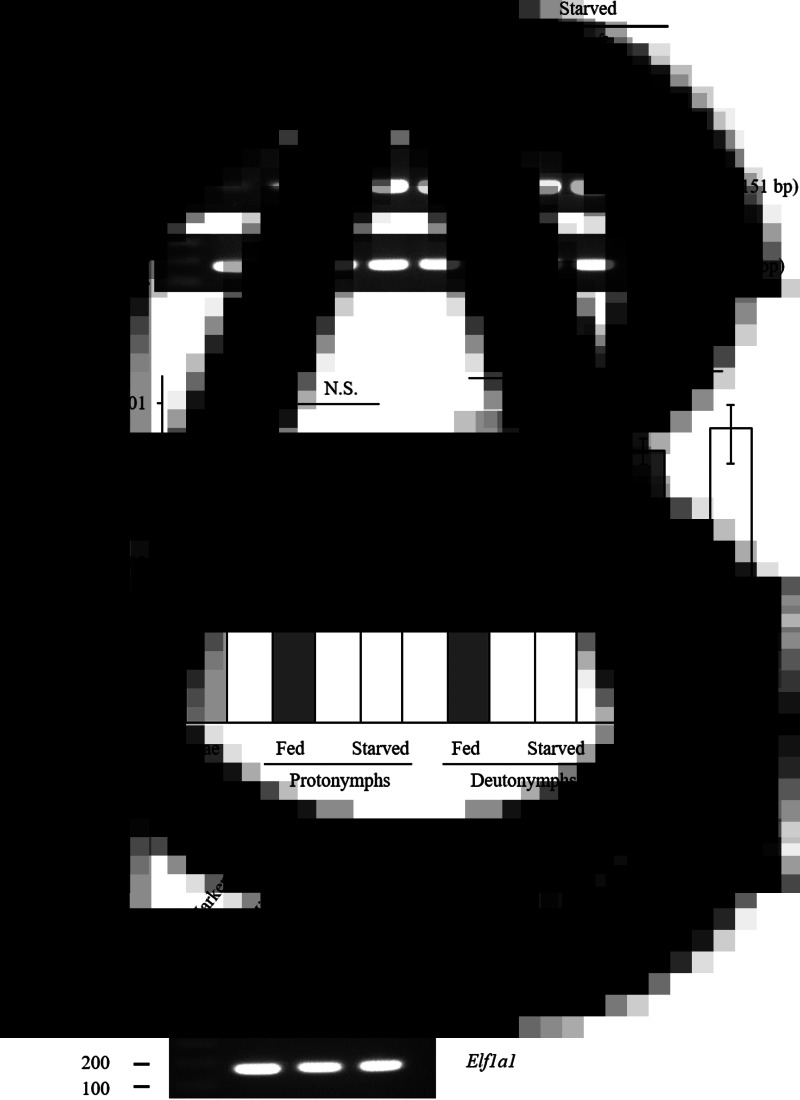
Gene-expression analysis of *Dg-Ctr1*. *Dg-Ctr1* expression was examined by RT-PCR/nested PCR at each life-stage and blood-feeding states of PRMs (A), and different tissues of PRMs (C). *Elongation factor 1-alpha 1*-like gene (*Ef1a1*) was amplified as an internal control. (B) Real-time quantitative RT-PCR was performed to quantify the gene expression of *Dg-Ctr1* at each life-stage and blood-feeding state of PRMs. The extent of *Dg-Ctr1* expression was calculated by dividing the copy numbers of *Dg-Ctr1* by those of *Elf1a1*. Each experiment was repeated four times and error bars indicate s.e.m.. Statistical analyses were performed using Mann–Whitney *U* test. (C) The expression of *Dg-Ctr1* in the midguts and ovaries was analysed by RT-PCR, and the expression in the salivary glands was analysed by RT-nested PCR.

The gene-expression profiles of *Dg-Ctr1* were analysed, and the expression of *Dg-Ctr1* mRNA in different life stages was examined. RT-PCR analysis revealed that *Dg-Ctr1* mRNA was expressed in all the life stages (Fig. 2A). Moreover, qRT-PCR analysis showed no significant difference in the expression level of *Dg-Ctr1* mRNA between blood-fed and starved PRMs, in contrast to the data from the RNA-Seq analysis (Fig. 2B, Supplemental Table 1). To examine the expression of *Dg-Ctr1* in different tissues, LCM and RT-PCR/nested PCR were performed using tissue samples from midguts, ovaries and salivary glands. *Dg-Ctr1* expression was clearly observed in the midguts and ovaries, while it was not detectable in salivary glands (Fig. 2C). Collectively, *Dg-Ctr1* seemed to be constitutively expressed in all the life stages, and importantly, it was expressed in the midguts and ovaries.

### Expression of Dg-Ctr1 on the plasma membrane

**Fig. 3.**
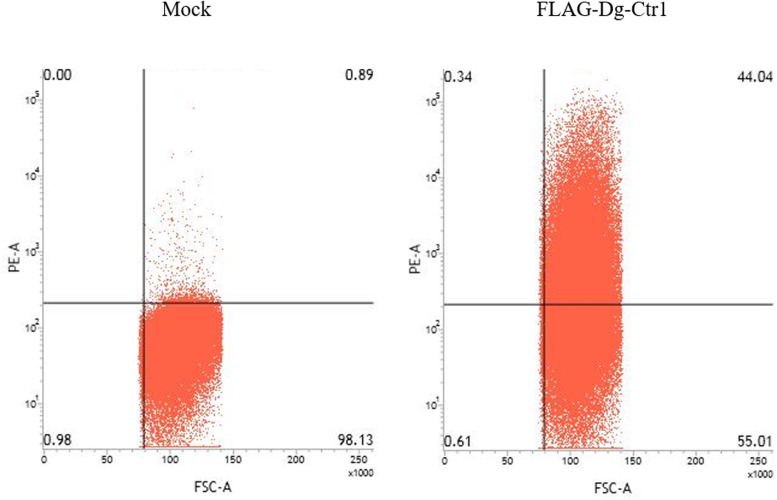
Expression of Dg-Ctr1 on the plasma membrane. FLAG epitope-tagged recombinant protein of Dg-Ctr1 (FLAG-Dg-Ctr1) was expressed on Sf9 insect cells. The expression of Dg-Ctr1 on the plasma membrane was confirmed by flow cytometric analysis using anti-DYKDDDDK (FLAG) antibody.

The N-terminal FLAG epitope-tagged recombinant protein of Dg-Ctr1 (FLAG-Dg-Ctr1) was expressed in the Sf9 insect cells, and the subcellular localization of Dg-Ctr1 was examined by flow cytometric analysis using the anti-DYKDDDDK (FLAG) antibody. In comparison with mock-transfected cells, FLAG-Dg-Ctr1-expressing cells were more frequently recognized by the anti-FLAG antibody (Fig. 3), suggesting that Dg-Ctr1 was a plasma membrane protein, as predicted by InterProScan.

### Acaricidal potential of plasma from chickens immunized with Dg-Ctr-N-his

**Fig. 4.**
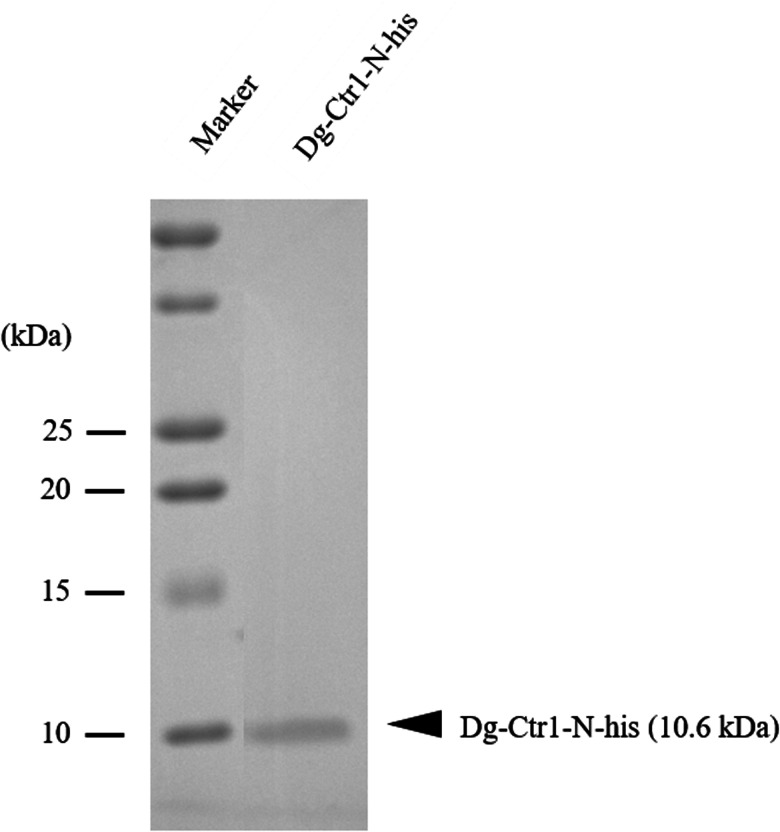
Preparation of recombinant Dg-Ctr1. The recombinant protein of N-terminal extracellular domain of Dg-Ctr1 (Dg-Ctr1-N-his) was prepared using BIC system. Purified Dg-Ctr1-N-his was separated by SDS-PAGE and visualized by staining with Coomassie brilliant blue.

**Fig. 5.**
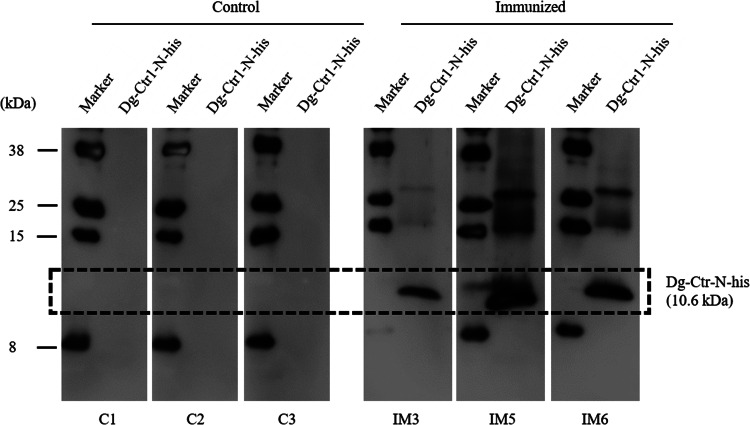
Dg-Ctr1-N-his-specific antibody production of immunized chickens. The recombinant Dg-Ctr1-N-his was reacted with the plasmas from immunized chickens (IM3, IM5 and IM6) and control chickens (C1, C2 and C3) by western blotting. The predicted molecular size of Dg-Ctr1-N-his is approximately 10.6 kDa, and the specific signals were detected only in the plasmas from the immunized chickens.

**Fig. 6.**
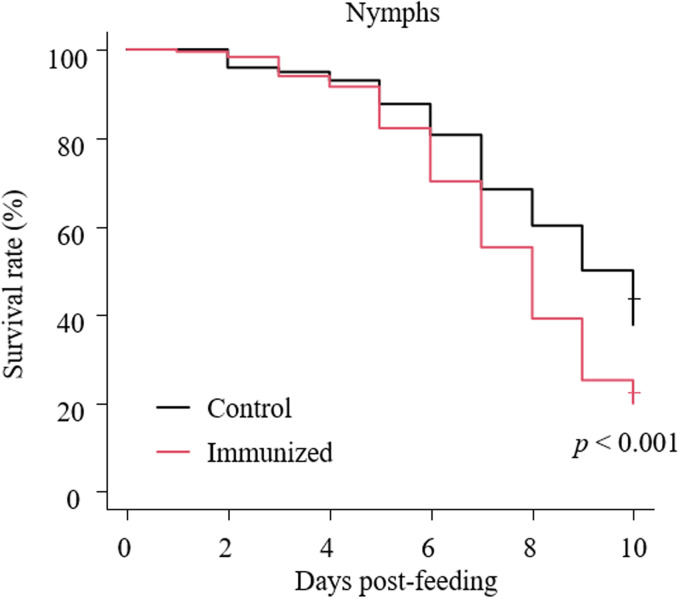
Anti-PRM effects of plasma from chickens immunized with Dg-Ctr1-N-his (first experiment). The survival rate of PRMs that were fed the plasma from immunized chickens was assessed every day for 10 days. The Kaplan−Meier curves was generated to indicate the survival rate in PRMs. Statistical analyses were performed using log-rank test. *P* < 0.01 was considered statistically significant.

**Fig. 7.**
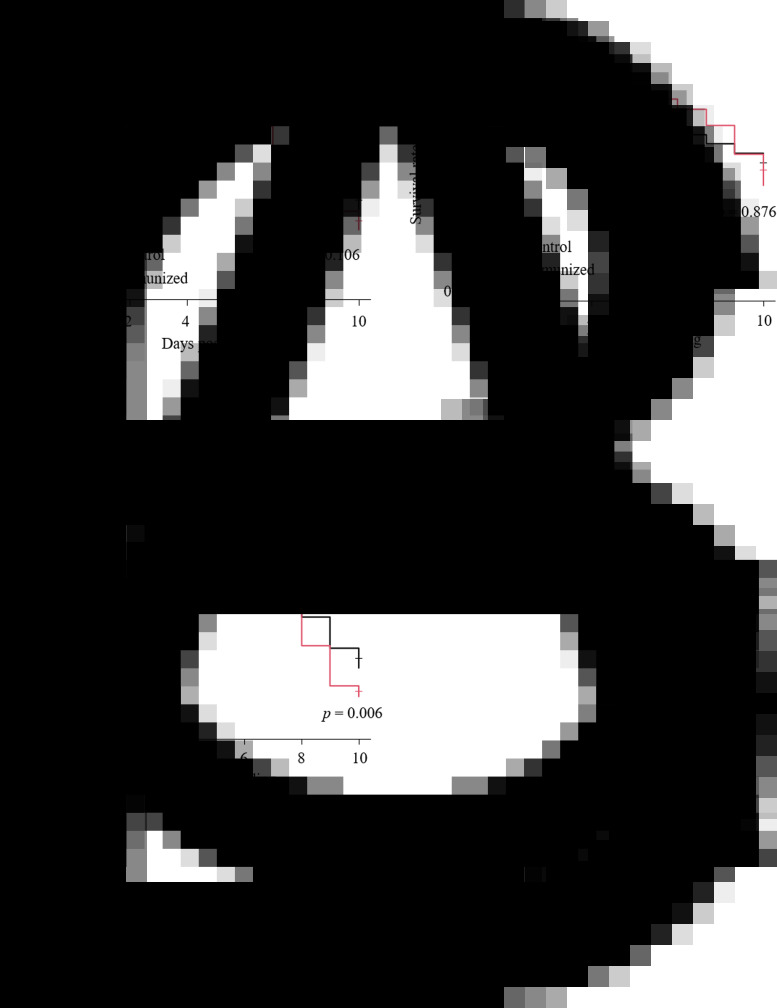
Anti-PRM effects of plasma from chickens immunized with Dg-Ctr1-N-his (second experiment). The survival rate of PRMs that were fed with the plasma from immunized chickens was assessed every day for 10 days. (A–C) The Kaplan−Meier curves was generated to indicate the survival rate in total PRMs (A), adults (B) and nymphs (C). Statistical analyses were performed using log-rank test. *P* < 0.01 was considered statistically significant.

The recombinant protein of the N-terminal extracellular domain of Dg-Ctr1 was prepared as a fusion protein with His-tag (Dg-Ctr1-N-his) using the BIC system. Dg-Ctr1-N-his was purified from the culture supernatant of the transformed bacteria, and the expression and purity of Dg-Ctr1-N-his were confirmed by SDS-PAGE and by staining with Coomassie brilliant blue. Dg-Ctr1-N-his was detected at the predicted molecular weight (approximately 10.6 kDa) (Fig. 4). Chickens were subcutaneously immunized twice with Dg-Ctr1-N-his in an emulsion with light liquid paraffin, and the plasmas were collected according to the schedule described in Supplemental Fig. 1. The antibody titres in the immune plasma were determined by ELISA and chickens IM3, IM5 and IM6 exhibited higher antibody titres against Dg-Ctr1-N-his (Table 2). In addition, western blot analysis revealed that the antibodies produced in IM3, IM5 and IM6 included antibodies specific against Dg-Ctr1-N-his (Fig. 5). The plasmas from IM3, IM5 and IM6 and plasmas from three chickens of the control group were used in the *in vitro* feeding assay to evaluate acaricidal effects, as described previously (Ariizumi *et al*., 2021). After 4 h of the *in vitro* feeding, blood-fed PRMs were collected, and the number of blood-fed PRMs collected and their life-stages were counted. To assess the acaricidal potential, the survival rate of PRMs fed on the immune plasmas was monitored. In the first experiment, only nymphs were obtained, and the Kaplan–Meier curve analysis and the log-rank test indicated that PRMs fed on the plasmas from IM3, IM5 and IM6, chickens immunized with Dg-Ctr1-N-his, showed a significant reduction in survival rate (Fig. 6). Additionally, Fisher's exact test and odds ratio analysis showed that the survival rate of PRMs fed on Dg-Ctr-N-his-immunized plasmas decreased from 7 days post-feeding (Table 3). In the second experiment, Fisher's exact test and odds ratio analysis showed that the survival rate of PRMs fed on immune plasmas was significantly lower than that of control groups at 10 days post-feeding when PRMs of mixed life stages were assessed, whereas no significant difference in the survival rate was indicated by the log-rank test (Fig. 7A, Table 4). To assess the effects of immune plasmas on PRMs of different life stages, nymphs and adults were analysed separately. For adults, neither log-rank test or Fisher's exact test indicated significant differences in the survival rates between the control and immunized groups (Fig. 7B and Table 5). In contrast, consistent with the results of the first experiment, Kaplan–Meier curve and log-rank test revealed that the survival rate of nymphs fed with Dg-Ctr1-N-his-immunized plasmas was significantly lower than that of the control groups (Fig. 7C), and this decrease in the survival rate was observed 8–10 days post-feeding (Table 6). These results suggest that the plasmas from chickens immunized with Dg-Ctr1-N-his have the potential to increase the mortality of PRMs, especially nymphs. Thus, Dg-Ctr1 could be one of the candidates for vaccine antigens to effectively control PRMs.

**Table 2.** Antibody titres in the plasma from chickens immunized with Dg-Ctr1-N-his

Group	Chicken	Antibody titre
Control	C1	<1000
	C2	<1000
	C3	<1000
Immunized		
10 *μ*g	IM1	<1000
	IM2	4000
	IM3	32 000
20 *μ*g	IM4	4000
	IM5	> 64 000
	IM6	> 64 000

**Table 3.** Summary of the anti-PRM effects of plasma from chickens immunized with Dg-Ctr1-N-his (First experiment)

	Days post-feeding									
	1	2	3	4	5	6	7	8	9	10
Immunized (*n = 167, nymphs)*										
No. of dead PRMs	1	3	10	14	30	50	75	102	125	134
Survival rate (%)	99.40	98.20	94.01	91.62	82.04	70.06	55.09	38.92	25.15	19.76
*Control (n = 98, nymphs)*										
No. of dead PRMs	0	4	5	7	12	19	31	39	49	61
Survival rate (%)	100	95.92	94.90	92.86	87.76	80.61	68.37	60.20	50.00	37.76
*P* value	1.000	0.429	1.000	0.817	0.296	0.061	0.037*	0.0009*	5.40 × 10^−5^*	0.0023*
Odds ratio	–	0.43	1.18	1.19	1.57	1.77	1.76	2.37	2.96	2.45
95% CI (lower limit)	–	0.06	0.36	0.43	0.73	0.94	1.01	1.38	1.70	1.35
95% CI (upper limit)	–	2.61	4.55	3.62	3.55	3.44	3.09	4.09	5.22	4.47

PRMs were fed on plasma derived from chickens immunized with Dg-Ctr1 of PBS *in vitro*. After blood feeding for 4 h, nymphs were collected, and their survival was monitored for 10 days. The data were compared by Fisher's exact test between immunized and control groups.

*P < 0.05 was considered statistically significant.

**Table 4.** Summary of the anti-PRM effects of plasma from chickens immunized with Dg-Ctr1-N-his (Second experiment, total)

	Days post-feeding									
	1	2	3	4	5	6	7	8	9	10
*Immunized (n = 252)*										
No. of dead PRMs	2	13	20	26	52	87	110	137	173	192
Survival rate (%)	99.21	94.84	92.06	89.68	79.37	65.48	56.35	45.63	31.35	23.81
*Control (n = 241)*										
No. of dead PRMs	6	16	26	33	47	74	102	122	145	164
Survival rate (%)	97.51	93.36	89.21	86.31	80.50	69.29	57.68	49.38	39.83	31.95
*P* value	0.168	0.567	0.283	0.269	0.822	0.388	0.785	0.418	0.060	0.045*
Odds ratio	0.31	0.77	0.71	0.73	1.07	1.19	1.06	1.16	1.45	1.50
95% CI (lower limit)	0.03	0.33	0.37	0.40	0.67	0.80	0.73	0.80	0.98	0.99
95% CI (upper limit)	1.78	1.74	1.37	1.30	1.71	1.77	1.53	1.68	2.14	2.28

PRMs were fed on plasma derived from chickens immunized with Dg-Ctr1 of PBS *in vitro*. After blood feeding for 4 h, the adults and nymphs were collected and separated. Their survival rates were monitored for 10 days. The results of adults and nymphs were pooled. The data were compared by Fisher's extract test between immunized and control groups.

*P < 0.05 was considered statistically significant.

**Table 5.** Summary of the anti-PRM effects of plasma from chickens immunized with Dg-Ctr1-N-his (Second experiment, adults)

	Days post-feeding									
	1	2	3	4	5	6	7	8	9	10
Immunized (*n = 95)*										
No. of dead PRMs	1	7	9	10	15	22	26	32	43	55
Survival rate (%)	98.95	92.63	90.53	89.47	84.21	76.84	72.63	66.32	54.74	42.11
*Control (n = 80)*										
No. of dead PRMs	1	9	13	17	21	22	30	33	36	42
Survival rate (%)	98.75	88.75	83.75	78.75	73.75	72.50	62.50	58.75	55.00	47.50
*P* value	1.000	0.436	0.252	0.059	0.095	0.600	0.193	0.347	1.000	0.542
Odds ratio	0.84	0.63	0.54	0.44	0.53	0.80	0.63	0.72	1.01	1.24
95% CI (lower limit)	0.01	0.19	0.19	0.17	0.23	0.38	0.32	0.37	0.53	0.65
95% CI (upper limit)	66.76	2.01	1.46	1.09	1.18	1.67	1.25	1.40	1.92	2.36

PRMs were fed on plasma derived from chickens immunized with Dg-Ctr1 of PBS *in vitro*. After blood feeding for 4 h, adults were collected, and their survival rate was monitored for 10 days. The data were compared by Fisher's extract test between immunized and control groups.

**Table 6.** Summary of the anti-PRM effects of plasma from chickens immunized with Dg-Ctr1-N-his (second experiment, nymphs)

	Days post-feeding									
	1	2	3	4	5	6	7	8	9	10
*Immunized (n = 157)*										
No. of dead PRMs	1	6	11	16	37	65	84	105	130	137
Survival rate (%)	99.36	96.18	92.99	89.81	76.43	58.60	46.50	33.12	17.20	12.74
*Control (n = 161)*										
No. of dead PRMs	5	7	13	16	26	52	72	89	109	122
Survival rate (%)	96.89	95.65	91.93	90.06	83.85	67.70	55.28	44.72	32.30	24.22
*P* value	0.215	1.000	0.833	1.000	0.121	0.104	0.145	0.039*	0.002*	0.009*
Odds ratio	0.20	0.87	0.86	1.03	1.60	1.48	1.42	1.63	2.29	2.18
95% CI (lower limit)	0.00	0.24	0.34	0.46	0.88	0.91	0.89	1.01	1.31	1.17
95% CI (upper limit)	1.82	3.12	2.15	2.29	2.92	2.40	2.27	2.64	4.07	4.18

PRMs were fed on plasmas derived from chickens immunized with Dg-Ctr1 of PBS *in vitro*. After blood feeding for 4 h, nymphs were collected, and the survival rate was monitored for 10 days. The data were compared by Fisher's extract test between immunized and control groups.

*P < 0.05 was considered statistically significant.

## Discussion

Previous reports have demonstrated that copper ions play pivotal roles in arthropod physiologies by mediating biological activities, including respiration, pigmentation and immunity (Muttkowski, 1921; Christensen *et al*., 2005; Lu *et al*., 2014; Sugumaran and Barek, 2016), and have reported the acaricidal effects of synthetic copper-based nanoparticles on ticks since excess copper ion is toxic (Ramyadevi *et al*., 2011; Ingle *et al*., 2014). Thus, disruption of copper metabolism affects the mortality of arthropods and could be a target to develop a novel strategy for controlling haematophagous arthropods. The copper transporter 1 is expressed on the plasma membrane and is required for uptake of copper ions. Several studies on anti-tick vaccines have shown the acaricidal effect of vaccines targeting the cell membrane protein BM86 (Rand *et al*., 1989; Azhahianambi *et al*., 2009). Therefore, copper transporter 1, which is expressed on plasma membrane and related to copper uptake, can be considered to be a suitable vaccine antigen to control haematophagous arthropods. In the present study, a newly identified copper transporter 1-like molecule in PRMs, Dg-Ctr1 was characterized, and its potential as a vaccine antigen against PRMs was assessed.

The RNA-seq data in a previous study suggested that Dg-Ctr1 showed marked expression in both fed and starved PRMs (Fujisawa *et al*., 2020). On the basis of the deduced amino acid sequences, Dg-Ctr1 was predicted to possess three transmembrane domains and an extracellular domain at the N-terminal region, and Dg-Ctr1 was detected on the plasma membrane by flow cytometric analysis. In addition, gene-expression analyses revealed that *Dg-Ctr1* was expressed in the midgut and ovaries, regardless of feeding state and life stage. BM86 has been identified as a midgut glycoprotein expressed on the plasma membrane (Rand *et al*., 1989; Azhahianambi *et al*., 2009), and Dg-Ctr1 was considered to be a protein expressed in the midgut and on the plasma membrane. Thus, Dg-Ctr1 met the criteria for vaccine antigens against PRMs.

In the present study, no significant difference was observed in the gene-expression levels of *Dg-Ctr1* in each feeding state in PRMs of all the life stages. In contrast, the RNA-seq data showed that the expression intensity of the *Dg-Ctr1* transcript in starved PRMs was significantly higher than that in fed PRMs. RNA-seq analysis was performed using starved PRMs that were kept for a 2-week period without blood meals, while PRMs incubated without blood meals for a 1-week period were subjected to the qRT-PCR analysis in this study; thus, the difference in the expression patterns of *Dg-Ctr1* may depend on the period of starvation. In addition, several studies have reported that the membrane expression of Ctr1 in humans, yeast, and *Arabidopsis thaliana* were down-regulated in response to copper stimulation to strictly regulate copper homeostasis (Petris *et al*., 2003; Liu *et al*., 2007; Li *et al*., 2020). Thus, the expression levels of Dg-Ctr1 may change depending on the period after blood feeding. In this study, the expression level of *Dg-Ctr1* in blood-fed PRMs was compared with that in PRMs collected 1 week after blood feeding. To determine the gene-expression kinetics of Dg-Ctr1, therefore, its expression level should be examined at several time points after blood feeding.

In this study, we observed differences in the antibody response of immunized chickens. Considering the practical applications, future investigations should focus on dosing, routes and adjuvant use to improve the antibody response to immunization. In addition, to determine the optimal amounts of antigens, we need to examine the minimal antibody titre required to elicit the acaricidal effects.

To assess the potential of Dg-Ctr1 as a vaccine antigen, the survival rate of PRMs fed on the plasmas obtained from chickens immunized with the recombinant Dg-Ctr1-N-his was analysed *in vitro*. Significant reduction in the survival rate of PRMs was observed both in experiments 1 and 2. However, the acaricidal effects of the immune plasmas were different between nymphs and adults; nymphs exhibited an increase in mortality, whereas the acaricidal effect was not observed in adults. Although the underlying mechanism causing this difference in acaricidal effects between adults and nymphs remains unclear, the susceptibility to copper toxicity may differ between adults and nymphs. To elucidate the physiological importance of Dg-Ctr1 in each life stage, the functions of Dg-Ctr1 in adults and nymphs should be investigated using gene silencing experiments using RNA interference (Chen *et al*., 2021).

Taken together, the findings of the present study suggest that vaccination with Dg-Ctr1 could help control the number of PRMs in poultry farms. To the best of our knowledge, this is the first study reporting the potential effectiveness of a PRM control strategy targeting copper homeostasis. However, the acaricidal effect was restricted in nymphs and was observed from 7 or 8 days post-feeding, which is relatively later than the effects of other previously reported vaccine candidates (Bartley *et al*., 2012; Xu *et al*., 2020; Murata *et al*., 2021). Since PRMs feed on blood every 2–4 days (Sparagano *et al*., 2014), the acaricidal effects should emerge quickly. Importantly, PRMs were fed blood just once in our *in vitro* feeding assay. In the field, PRMs were fed blood over and over and were therefore exposed to antibodies more frequently. Therefore, to further evaluate the anti-PRM potential, PRM-infestation trial using Dg-Ctr1-immunized chickens should be examined. Additionally, to improve the efficacy of vaccines targeting Dg-Ctr1, the effects of its combined use with chemical acaricides or other candidates for vaccine antigens as ‘cocktail vaccines’ should be examined.

## Supplementary material

For supplementary material accompanying this paper visit https://doi.org/10.1017/S0031182021001608. click here to view supplementary material

## Data

All datasets are available in the main manuscript.
