# Supplementary material for: Characterization of a copper transporter 1 from Dermanyssus gallinae as a vaccine antigen
Source: Parasitology. 2021 Sep 10;149(1):105–15. doi: 10.1017/S0031182021001608 (PMC8862010; doi:10.1017/S0031182021001608)
Supplement: Supplementary file 1 [file S0031182021001608sup.zip › S0031182021001608sup002.docx]

**Supplemental table 1.** Gene expression profiles of a *Dermanyssus gallinae* copper transporter 1-like molecule (*Dg-Ctr1*)

|  | FPKM value^(a)^ | | Predicted motifs/domains^(b)^ | *p* value | FDR^(c)^ |
| --- | --- | --- | --- | --- | --- |
|  | Blood-fed | Starved |  |  |  |
| *Dg-Ctr1* | 102.5 | 373.99 | Ctr copper transporter | 0.023 | 0.050 |

Indicated data were adapted from the RNA-Seq analysis data presented by Fujisawa *et al* (2020).

(a) Fragment per kilobase of exon per million mapped reads

(b) The motifs/domains were predicted using the InterProScan program v5.32-71.0.

(c) False discovery rate

(age) 0 w 3 w 7 w 10 w

1st immunization

(10 or 20 µg/shot, s.c.)

2nd immunization

(10 or 20 µg/shot, s.c.)

Exsanguination

- Antigen : Dg-Ctr1-N-his, liquid paraffin inclusion (*n* = 3, each)
- Control : PBS with liquid paraffin (*n* = 3)

**Supplemental figure 1.** The schedule of immunization with Dg-Ctr1-N-his and sample collection
