# Supplementary figures and images for: Characterization of a copper transporter 1 from Dermanyssus gallinae as a vaccine antigen
Source: Parasitology. 2021 Sep 10;149(1):105–15. doi: 10.1017/S0031182021001608 (PMC8862010; doi:10.1017/S0031182021001608)

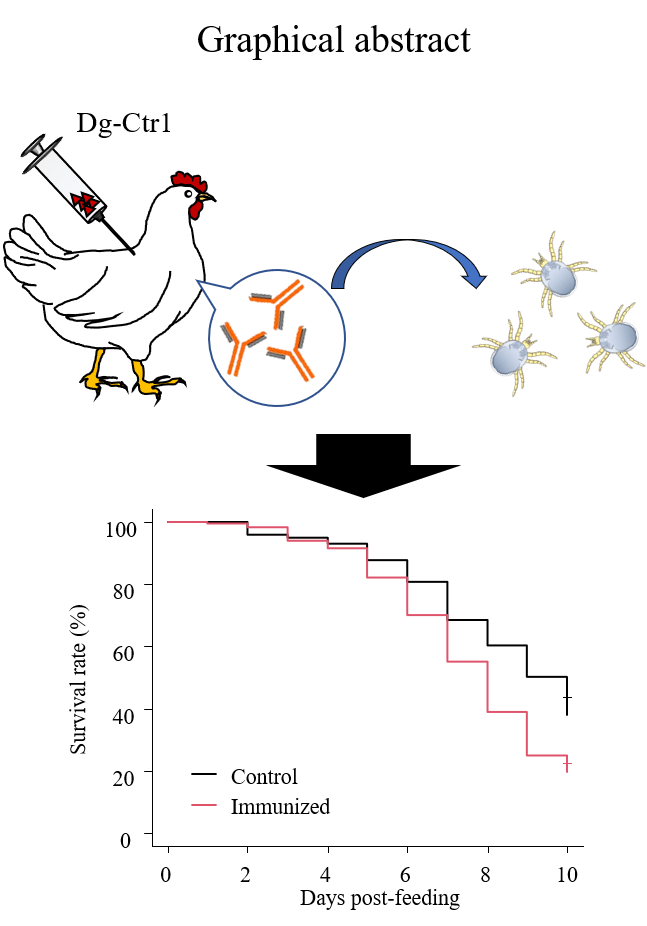

Supplement: Supplementary file 1 [file S0031182021001608sup.zip › S0031182021001608sup001.tif]
